# Perception, Attitudes, and Experiences Regarding Mental Health Problems and Web Based Mental Health Information Amongst Young People with and without Migration Background in Germany. A Qualitative Study

**DOI:** 10.3390/ijerph18010081

**Published:** 2020-12-24

**Authors:** Ümran Sema Seven, Mendy Stoll, Dennis Dubbert, Christian Kohls, Petra Werner, Elke Kalbe

**Affiliations:** 1 Medical Psychology, Neuropsychology and Gender Studies and Center for Neuropsychological Diagnostics and Intervention (CeNDI), Faculty of Medicine and University Hospital Cologne, University of Cologne, 50923 Cologne, Germany; elke.kalbe@uk-koeln.de; 2 Faculty of Information Science and Communication Studies, TH Köln/University of Applied Sciences, 50678 Cologne, Germany; mendy.stoll@th-koeln.de (M.S.); petra.werner@th-koeln.de (P.W.); 3 Faculty of Computer Science and Engineering Science, TH Köln/University of Applied Sciences, 50678 Cologne, Germany; dennis.dubbert@th-koeln.de (D.D.); christian.kohls@th-koeln.de (C.K.)

**Keywords:** adolescents, young adults, migrant background, mental illness, health literacy, internet/web information, focus group discussions, qualitative content analysis

## Abstract

Mental illnesses in adolescence and young adulthood are steadily increasing. Thus, mental disorders represent an individual and societal challenge and an enormous health economic burden, creating an urgent need for research and action. Mental health problems are omnipresent in the life of young people and the internet is the first resource, which helps them to understand their situation. Young people with migration background often have more difficulties accessing health care services. Digital technologies offer an ideal opportunity for a low-threshold platform that addresses the needs of young people. The current project “GeKo:mental” aims to design a multilingual website for Cologne-based adolescents and young adults that will enable them to obtain comprehensive information about mental illness and health, treatment options and first contact points. To design this website, this study aims to find out what kind of health information is needed and how it should best be presented. Nine focus group discussions with adolescents and young adults with and without migration background (N = 68) were conducted; the focus group discussions took place at schools, in an association for social youth work and in an cultural association, which is linked to a mosque in Cologne, Germany. A qualitative content analysis was conducted on the gathered material. The participants reported concrete challenges and needs. The results will form the basis for the development and design of a website.

## 1. Introduction

Nearly one quarter of Germany’s population of 83 million has a migration background. Adolescents and young adults aged 15 to 30 years make up about 13 million with 4.5 million having a migration background [1].

Mental illnesses in adolescence and young adulthood are steadily increasing [2]. Based on data from a health insurance company, in Germany mental illnesses rose by 38% to 1.9 million between 2005 and 2016 [3]. Adolescents are particularly vulnerable to mental health issues, especially to internet-related addictions such as computer and smartphone addiction, which is positively associated with anxiety, depression, and loneliness [4,5]. Depression is an important risk factor for suicide [6], which is the second leading cause of death in adolescents and young adults [7]. Many children and adolescents who have a significant mental health disorder have a higher probability to experience negative life developments [8]. Moreover, they are more likely to suffer from social isolation and stigmatization [9,10]. In general, people with severe and chronic mental illnesses have a significantly reduced life expectancy compared to the general population [11]. Thus, mental disorders represent an individual and societal challenge and an enormous health economic burden [12], creating an urgent need for research and action. 

The World Health Organization (WHO) defines mental health as a state of well-being in which an individual can develop or realize his or her potential, cope with the normal stresses of life, work productively, and contribute to the community [13,14]. Certain factors may increase the risk of developing mental illnesses in adolescence. According to the WHO, which surveyed 11- to 15-year-old adolescents in Europe, mental well-being decreases with age; adolescents report nervousness, irritability, and sleep disorders, with girls showing poorer mental well-being than boys [15]. Research shows that lower socioeconomic status and a migration background predisposes young people to develop a mental health illness [5,16,17]. Furthermore, people with migration background have consistently poorer health literacy than people without migration background [18]. In the context of health care, health literacy is defined as the ability to access, understand, evaluate, and implement health-related information [19]. Thus, it refers to the knowledge and competence of the individual to meet health-related requirements, including making decisions to maintain and improve health and to deal with illnesses appropriately. This competence is determined by individual sociodemographic factors (e.g., age, gender, and ethnicity), situational factors (such as social support and media use) and societal or environmental factors (e.g., culture, language, and demographic situation) [19]. 

A migrant is a person who was not born as a German citizen or who has at least one parent to whom this applies. People who have a migration background differ from people of German origin in health issues due to specific health beliefs and behavior [18]. Due to several barriers, they often have more difficulties accessing health care services, which in turn can lead to a poorer state of health and different health-care utilization trends. Having access to health care and educational opportunities is also more difficult for young people with a migration background [20,21]. A study showed that young people with a two-sided migration background (i.e., both parents) [22] reported psychological problems more frequently (16.9%) than young people without a migration background (11.5%) or young people with a one-sided migration background (11.3%) [23]. Overall, the concept of health literacy has received little attention and a more specific overview of health literacy among young people is lacking [24]. There is evidence of a link between health literacy and the health behavior of young people, so there is a need to promote health literacy [25]. Initial evidence shows that adolescents have poorer health literacy than adults. For example, an Austrian study [26] found out that 68% of adolescents (versus 28% adults) have difficulties finding information about disease symptoms that affect them. Especially when it comes to mental health promotion, young people face difficulties in finding information. Previous studies have shown that low health literacy is associated with health and social consequences such as riskier health behavior, low utilization of prevention services, delayed diagnosis and treatment, lower compliance, and poorer physical and mental health [27,28]. Missing or late diagnosis can lead to significantly worse outcomes, especially in the case of mental illnesses. Evidence shows that particularly young people with a migration background have inadequate health literacy [29].

Health socialization takes place in adolescence [29], which raises the question what kind of sources young people use to obtain health-related information. Nowadays, digital media play an important role in everyday life [30,31]. The “Digital Index” (scale from 0 to 100) indicates the degree of digitization in society, summarizing the components access, competence, use, and openness. In Germany, the average Digital Index is 58, in which the age group of 14- to 29-year-olds reaches the highest value of 73 points [32]. Therefore, target group-specific web offerings are required to reach young people and make mental health-related information widely accessible. 

Individuals who experience mental health issues may have feelings of shame and fear stigmatization from their family, friends, or society. Young people may be reluctant to ask for help or to get treatment and are therefore likely to isolate themselves from the world. That is why it is important to provide digital opportunities in which young people themselves search for information. However, it is important to address the dangers of the internet as well, as non-serious sources can be accessed by those affected [33]. For example, false information can circulate on the Internet, which can also be triggering. In addition online offerings that address psychological topics are not exclusively aimed at young people, and the target group of young people with a migration background is hardly addressed at all. 

In recent years, an increasing number of people have migrated to Germany with most of the applicants being younger than 30 years old [34]. The migration experience can be a stressful process; hence, the migrant population is more vulnerable to have mental disorders due to traumatic experiences and they may experience post-traumatic stress disorder, adaptation problems, anxiety, and depression [35]. Nevertheless, there is a lack of adequate information and treatment options available. Consequently, offering insight to mental illnesses and the promotion of mental health through digital sources have a high relevance particularly for vulnerable adolescents and young adults and especially for those with migration background. 

Jorm and colleagues introduced and shaped the construct of Mental Health Literacy (MHL) [36] due to the neglect of mental disorders in the context of health literacy promotion [37]. The construct of MHL represents an extension of health literacy [36]. According to this construct, MHL comprises the ideas and knowledge about mental disorders, which in turn form the basis for recognition, coping, or prevention. It is characterized by seven components: ability to recognize specific mental disorders; knowledge of how to obtain mental health information; knowledge and beliefs regarding risk factors and causes; knowledge and beliefs regarding self-intervention strategies; knowledge and beliefs regarding available professional services; attitudes that promote recognition; and appropriate seeking of help [36,38].

Improving the target group’s ability of to recognize signs of mental disorders at an early stage, to classify symptoms correctly and to be able to obtain reliable information about contact points, could increase the motivation to seek help and treatment, what substantially leads to better mental health outcomes and a better quality of life in the long term. 

Digital technologies offer an ideal opportunity for a low-threshold platform that can be easily adapted to specific target groups for people with and without a migration background. The current project aims to design a multilingual website in German and English that will enable young people to obtain comprehensive, appealing, and comprehensible information about mental illness. To design this website according to the needs of young people, this study aims to find out what kind of health information is needed and how this information should best be presented. The main objectives of this study were to explore the presence and relevance of mental health problems in the life of young people with and without a migration background and how they deal with it, to conclude their health literacy. Focus groups were set up to examine, from the perspectives of young people, their experiences with mental health problems, challenges, and needs. The results should form the basis for the development and design of a website.

**Figure 1:**
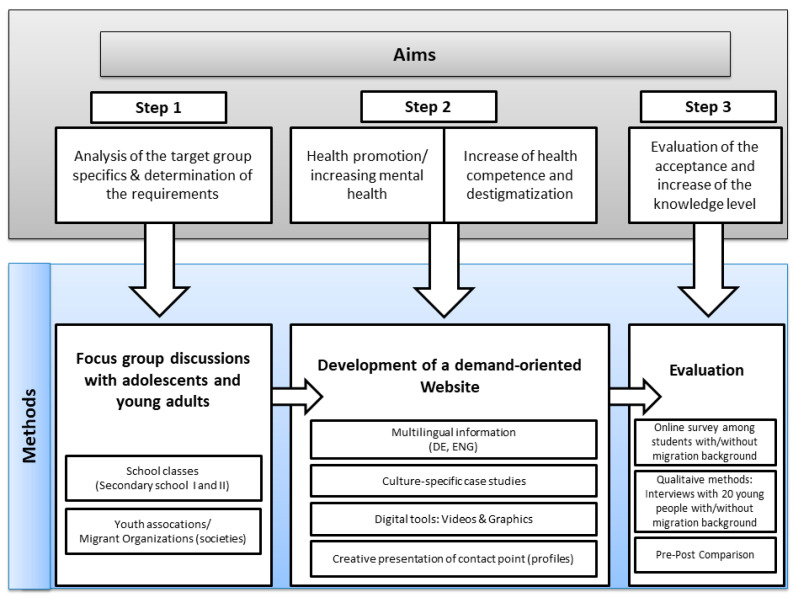
Overview about steps, aims, and methods of the research project.

With the development of a target group-oriented website, the following goals should be achieved:

- The health literacy for the mental health of adolescents and young adults with and without a migration background is to be promoted through the information on the website.
- The target group should be sensitized to the relevance of psychological problems and illnesses regarding themselves and their environment (peers), thus preventing chronification.
- Through the presentation of diverse case studies, an understanding of the individual approach to mental health and illness is achieved, which in turn leads to a reduction in stigmatizing behavior and an increase in the compatibility of the topic of mental health with the everyday life of young people.
- The website should provide an impression of trustworthiness and professionalism so that visitors are encouraged to deepen their knowledge about mental illnesses and are able to cope with previously challenging or overwhelming situations.
- In the long term, this should help to improve the health and quality of life of the target groups.
- This study refers mainly to Step 1 and summarizes ideas from its results that will be incorporated into Step 2 (see Figure 1).

## 2. Materials and Methods

### 2.1. Focus Group Discussions (FGD) and Guideline Development

Focus group discussions are often used in qualitative research and are structured by the application of a guideline. This guideline is developed for a specific research question to be able to capture its subject of interest [39]. Regardless of the fact that Focus Groups are relatively resource-saving [40], they were chosen because they are appropriate for the investigation of the reality of life, experiences, or opinions and are therefore particularly suitable for the exploration of health care research [41,42]. They have also proved to be successful in an intercultural context, which requires aspects of cultural sensitivity and understanding on the part of the moderator [43,44]. The structuring of the FGD by a guideline aims to enable the comparison of different FGD and at the same time the collection of a broad spectrum of opinions. To achieve this comparability, the FGD is guided by the moderator. At the beginning, the participants are told which aspects of the topic will be dealt with and how the FGD will proceed. However, variations of topics can ensure that a discussion among the participants takes place and the conversation does not fall silent prematurely. At best, the participants feel stimulated by the statements of others to deeper considerations and expressions of their own. The moderator has to recognize the group dynamics and give them free rein without losing sight of the focused topic.

The interview guideline was created by the research team based on previous research on relevant aspects (including research on existing websites on mental illness, and health literacy). In addition to an introductory question, several questions on the aspects of interest were formulated in order to loosely supervise the conversation or point towards left out topics which, in the end, lead to more detailed information. The questions refer to the following aspects:

- Everyday life of young people (challenges; the reality of life):
  The participants were generally asked how they were doing in everyday life, whether there are things at school, at work, or in the family that stress them. They were also asked how they deal with possible problems and challenges.
- Procurement and understanding of health-relevant information:
  It was discussed where and how the participants obtain information on health topics. In addition, specific information formats such as texts, videos, etc., were addressed in order to determine preferences, among other things.
- Importance of mental health (experiences and handling):
  Previous experiences with mental illnesses in the personal environment and general interest in psyche were discussed. In particular, the need for information and the evaluation of the information found with regard to comprehensibility and usefulness were addressed.
- Demands on digital services in general and in the context of mental health:
  Participants were asked for websites and apps that are used in everyday life. They were also asked what they like about these diverse digital possibilities and possibly also media people, e.g., on YouTube, and what they do not like and to what extent there is room for improvement. Of particular interest was the question of the seriousness, trustworthiness, and quality of online offerings. Additionally they were asked about their use of chat rooms or forums as well as quizzes or playful offers on websites.

The study was approved by the Ethics Committee of the Medical Faculty of the University of Cologne (n. 19-1192). The first FGD was conducted as a pretest. The pretest allows to test relevant aspects for the execution of a focus group. Testing the guideline is of primary importance, because it can provide information on whether the composition of the group is optimally aligned with the research questions and the goal of the project, and nevertheless because it offers the opportunity for the moderator to train techniques of discussion leadership in a real focus group situation. During the implementation of this first FGD no difficulties occurred and therefore there was no need for modifications regarding questions, settings, or moderator behavior. For this reason, the data of this FGD, originally announced as pretest, were the first to be included in the present analyses. In the present study nine FGD had to be conducted until saturation was reached, i.e., an additional group is not expected lead to substantially new results [45,46]. 

### 2.2. Participant Recruitment

Through our associated partners, the project researchers had the opportunity to advertise the project in schools and cultural associations (with special offers for young people) with posters. One school offered the possibility to take part in the FGD during a project week. Students contacted the teachers if they were interested and were given the information and consent forms to sign in advance. This procedure was used in four FGDs. At another school, the research project was also posted on notice boards for several weeks, so that students could contact the secretary’s office if they were interested. A similar procedure was implemented for the associations.

In order to obtain results relevant to the target group, the groups were kept as homogeneous as possible. In particular, these were groups that were more or less familiar with each other from the school or club context. The age range within the respective groups was relatively small. Male and female participants were represented in the FGDs that took place in schools. In the four groups that took place in association for social youth work and in a cultural association, only the last group with refugee participants had mixed genders. The other three groups were separated by gender, one purely male and two purely female. The participants were interested persons who responded to the study announcements in schools or at clubs. The participants from schools—a total of 5 constructed focus groups—were mixed in terms of age, migrant background, and gender, as befits everyday school life. The participants from the clubs were homogenous and natural groups [47], which means that the participants are connected to each other’s in everyday life. One of the female groups, a regular leisure group, consisted of Arabic participants from North Africa. The other female group, consisting of Turkish adolescents, meets regularly at the mosque and undertakes leisure activities. Another group consisted of 5 Turkish and one North African male participants, who already knew each other from previous meetings. Since the participants in this study were part of the study voluntarily (in the sense of self-recruitment), no statement can be made about how many people refused to participate. There were no participants who neither left focus group discussions nor subsequently revised participation. 

### 2.3. Implementation of the FGD

Before each group discussion, consent forms had to be signed and collected. For those under 18, a legal guardian had to sign in addition to the student him- or herself. Since, as already mentioned, the associated partners (teachers and association staff) handed out the information material and the declarations of consent to the interested parties in advance, there were only a few exceptions to the fact that the information material was read and signed before the FGD began. They were given sufficient time to read the material before signing the consent form. Despite these preparations, the information was repeated by the moderator before the start of the FGD and sociodemographic questionnaires with questions on gender, age, migration background, and current educational situation were distributed.

Two researchers and one assistant participated in every focus group. One person moderated the discussions, the other researcher documented relevant aspects for later recaps and spontaneously participated in the conversation if the situation required so. Here, the female researcher Ü.S.S. was in all focus groups on the role of the leading moderator. The assistant (psychology student) kept minutes of the speaker sequence in order to be able to assign the statements to the participants after transcribing the material. To avoid technical problems and to secure the recording, two audio recording devices were used.

The standardized procedure was as follows: The researchers welcomed the participants, introduced themselves and outlined the project plan. In order to start and maintain a positive atmosphere, the following aspects were asked to be considered: that the discussion remains confidential, that any opinion is welcome, that questions are allowed at any time, and that the focus group can be terminated at any time without giving reasons.

At the beginning, the participants were asked to introduce themselves and to give an introductory statement about what interests them about the topic and/or what motivates them to participate. The sound recordings were started before this round of introductions.

In order to actively promote the participants’ willingness to discuss, the moderator clarified open aspects by asking questions, verified the participants’ own understanding by paraphrasing, and in some cases made summaries to guide the course of the discussion.

As proof of their participation, the participants received a certificate from the University Hospital Cologne and the TH Köln University of Applied Sciences. In this certificate, the project and its goals were outlined and the focus group discussion was described, including information on how long it lasted. The participants were interested in receiving this certificate to use it in their CV as active voluntary support of a research project.

### 2.4. Data Analysis

After the focus groups had been carried out, the material, i.e., the tape recordings from two audio devices, were saved as files. One of the files was used as the basis for the transcription. The other file served as a backup file and was used when there were acoustic difficulties in understanding the spoken word. The discussions were transcribed verbatim (in German). After their completion, the analysis of the material was done with MAXQDA, a software for qualitative data analysis [48,49]. This research is based on the Consolidated criteria for reporting on qualitative studies [50].

For the qualitative content analysis, categories were formed both deductively and inductively [51]. This combined approach has advantages, among others: The deductive categories are based on the guideline and thus reflect the research questions, which serves their exploration. The development of inductive categories from the material makes it possible to generate new aspects that were not previously considered. 

The first two focus group transcripts (1 group with male participants with a migration background and 1 group with mixed-gender participants with and without a migration background) were coded by the researcher (Ü.S.S.) using MAXQDA (VERBI Software, Berlin, Germany). Here, the aspects of the interview guideline that follow the research questions served as the basis for forming the deductive categories. In addition, inductive categories were formed from the material. After completing the coding of the first two transcripts, another researcher (M.S.) checked the codings and the category system for traceability and completeness. After completion of this check of the analysis results, some subcategories were created and this slightly modified version was discussed and consolidated as a basis for the further coding. Based on this modified category system, Ü.S.S. coded the remaining seven transcripts. After the coding was completed, researcher M.S. again checked all the material for comprehensibility and completeness. There were no significant changes, so that the present analysis has been substantiated by two researchers, both of whom were also in the role of moderator and co-moderator. 

The quotations of the participants used in this manuscript were translated by a fluent English speaking researcher.

## 3. Results

Nine FGD with young people were conducted between July 2019 and February 2020. A total of 68 young adults participated in the FGD. Among them were 25 persons of male gender (36.8%) and 43 of female gender (63.2%). The average age of male participants was 23.8 years (SD = 3.2 years) and that of female participants was 22.5 years (SD = 4.0 years). There were 28 participants (41.2%) who had no migration background, whereas 40 participants (58.8%) had a migration background, which was recorded using a sociodemographic questionnaire. There was one group with only female participants of Arab origin (North Africa), and another group with only male participants of Turkish origin and one Arabic participant from North Africa. The second last FGD of a youth group of a mosque was only with female participants of Turkish origin. The FGD in schools were mixed gender and intercultural. The characteristics of the participants are presented in Table 1.

The participants showed great interest in the topic and usually spent 90 min discussing the aspects. Personal experiences with psychological topics were shared with the other participants in a confidential setting and very tangible ideas and wishes were expressed regarding digital offerings.

An overview of the categories formed is shown in Table 2. As already mentioned, these were generated deductively and inductively. The most important categories cover research questions relating to the everyday lives of young people, such as media use, challenges in everyday life and experiences with mental health problems. The general use of media and its importance were also surveyed, as well as media use for information needs on health issues. Following the model of Sorensen et al. [19], the coding of the material was also carried out with regard to the health literacy components. In the following, a selection of the results is presented as an example based on the participants’ quotes.

### 3.1. Experiences with Mental Illness

Many participants had experiences with mental health problems and were aware of similar experiences of relatives and friends. In the FGD, the participants consistently named various psychological symptoms and illnesses, including personality disorders such as borderline, eating disorders, self-harming behavior, sleep disorders, schizophrenia, anxiety disorders, depression, and suicidal tendencies. Three participants even reported about relatives or friends who committed suicide. The topic of mental stress and illness is omnipresent in the target group. There was a great willingness to talk about the topic. Answers range from every day, “little problems”, to many years of experience with mental illness. Some were satisfied with the treatment, others were very frustrated. Participants who have not yet had any connection to the topic were willing to inform themselves about it (“Sad to know so little”) or would have been grateful for an appropriate offer in case of a confrontation.

In terms of reasons were mentioned: “generational problems”, lack of planning; fast pace of life; dullness; social pressure, comparisons with others or “false” ideals (Instagram and celebrities), superficiality, bullying, “nobody reflects on themselves anymore”, anonymity, hardly any social contacts outside the social media; even in personal meetings “everybody is hanging on the cell phone.”

The majority of respondents expressed psychological problems as a relevant problem in their social environment and at the same time expressed the wish that this should receive more attention.

“I can imagine that every second person in this school has problems or knows people who have problems with something like that. I think it would be really helpful if there was a psychology subject in school. Then you already learn about it in young age. You would be able to help those affected in some way.”

(female, 18 years old, with migration background (wMB)

“Well, I just wanted to say again that I am in favor of everyone seeing a psychologist. And I also think that you should not necessarily call it a psychologist, because the word is always a bit negatively afflicted with wrong ideas. Instead, everyone should be entitled to a kind of life coach. This person should of course have a professional training, because you can get a lot out of personalities if you work on them for a bit. It doesn’t necessarily always have to be that someone only has one blatant problem but that you also find something else to work on.”

(female, 29 years old, without migration background)

#### 3.1.1. Supply Deficits and Consequences

The participants reported almost unanimously from their own experiences or those of their relatives that it is problematic to get a psychotherapy place. Both the procedure in general of overcoming oneself and finding someone and the fact that it takes a long time to get a therapy place.

“If you have to go to a psychologist or something like this, you often have to go to a general practitioner first to get a medical certificate. Additionally you have to wait for months until you get an appointment. And people often need acute care. Sometimes it is really urgent, and I think in this case using the internet as an interim measure until an appointment is actually made—there is nothing wrong with that. But as I just said, it is often the case that during this time so many influences can have a negative impact, that it is sometimes too late…three months ago it was too late for a friend of mine and she threw herself in front of a train. She waited. She had an appointment…and it was so acute and yes, that’s when she decided to commit suicide. And I simply believe an interim solution definitely has to be provided.”

(female, 28 years old, without migration background)

“I have a friend who came to see me last week. And one night he got some bad news: His friend had killed himself. He is from Afghanistan, and his asylum application has been rejected. And I think he had been in Germany for four or five years. My friend told me, ‘Yes, he suffers from depression.’ For already, I think, a year or something like that. […]. For example, ‘I feel very bad and I need someone who always listens.’ But my friend told me, ‘You know what, when you listen to him, you feel bad yourself.’ And because of that, no one wants to help him. Because there’s a bad feeling coming back. But after he had killed himself, all friends were like, ‘We wish we could listen to him just once more one time,’ and things like this. But now it’s all over and… I think that these people, who suffer from depressions, always need someone who listens and comforts. But I think refugees have no one like that, and that’s why it´s so hard.”

(male, 23 years old, with migration background and refugee experience)

#### 3.1.2. Attitudes and Modes of Behavior

Some participants expressed that the topic of the psyche is more present in society, but admitted that it is still repressed and is hardly ever talked about in school or in private contexts.

Parents usually do not want to believe that their child has psychological problems. Experiences were reported in which parents adopted a defensive attitude and also related the presence of a psychological problem personally to their upbringing: “That can’t be. That is not the way we raised our child. It has to be healthy.” Parents see external circumstances such as having a good life as a guarantee for physical well-being. It was also mentioned that parents generally lack understanding, and that psychological illnesses or symptoms that limit active participation in life (especially school or job) are associated with laziness. A consequence that relatives who don’t understand or simply underestimate mental problems is that the affected children lose the courage to turn to others, e.g., professionals, because they expect the same unsupportive, indifferent and questioning attitude.

The experience of a lack of understanding within the family often leads to frustration and resignation and is subsequently generalized:

“In some cultures… there is simply no such thing. So as soon as you start talking about not being well, even though there is no particular reason or you have dark thoughts or even think about death, they you say, ‘That’s bullshit. You are fantasizing it. You are simply imagining it to yourself. That is not true and so on. You have everything.’ You are accused of such things…. If you are already introverted and sad anyway, if you have depressions or something like that, then you tend not to say anything anymore. Also to friends or to doctors or somebody else. Because it’s your own parents. So if they already speak against you, what else should a doctor do.”

(female, 18 years old, with migration background)

“That’s exactly how it was with me. I didn’t tell my mother either and tried to do it myself, because I knew that she would say that I am just crazy. Of course I am crazy. that literally was the problem.”

(female, 29 years old, without migration background)

The question of whether mental illness is still a taboo subject today was answered very differently. Some said that nowadays people deal with them much more openly and that this is particularly evident in subcultures and in music. Comparisons were made with the past, but today there is greater acceptance and knowledge. It was emphasized even stronger that the topic has only rudimentarily arrived in society and should be given much more attention. The following quotation illustrates this need again:

“I personally think this topic is still too quiet. Far too quiet. And I think society needs to be sensitized much more to this topic. I also think that these people deserve to be heard and that they should not be treated with contempt. That they are taken seriously. And that people shouldn’t dismiss it and say, ‘Oh come on, he has his little ailments there’ or ‘But I also have my problems’.”

(female, 28 years old, without migration background)

There were also fears of rejection or criticism of one’s own person by others, as this person pointed out:

“Yes, you can also offer a target in some way. Even if you don’t expect to be attacked by your family or friends, but still.… You give something of yourself away.”

(female, 21 years old, without migration background)

#### 3.1.3. Problems with Knowledge Platforms and Mistrust on Social Media

The majority of participants use desktop PCs/laptops as well as smartphones and tablets. All participants use at least one social medium; the most common are:

- WhatsApp (communication medium; “You can’t do without it”; otherwise you are excluded; what makes it special is that it is free and fast)
- Instagram (serves as a distraction; but also: dangerous ideals are presented there—both in terms of body ideals and lifestyles such as vacation photos; “perfect”, exciting life)
- YouTube (both as “problem solver” and for fun)
- Facebook (named as a contrast to Instagram: Networking through groups, events and sales; criticism: flood of information that doesn’t interest you at all)

The opinions of the participants differ on the following points: Some said, “You would be happier without apps”, others tended in the direction of, “Apps make everything easier”. In addition, the overstimulation and waste of time was seen by some as critical and the observation was made that personal contact has decreased significantly.

The most important criterion for good media offerings in general is that new content is constantly being delivered. Regarding the planned website, it was said that it must be of a high quality compared to others.

Chat forums are viewed critically because “Anyone can answer and pretend to be a professional”. At the same time it was mentioned that trust could be increased by the hurdle of login. Nevertheless, communication “in private” is desired and proof that you are dealing with a professional person and anonymity is maintained.

“Well, I think such forums would make sense, because there is such a thing, where you can call, no matter what the topic is. There is still a bit of inhibition threshold, if you really call this anonymous number or not. And you don’t know who is on the phone right now. Does the person really understand you? Then sometimes it is perhaps easier to explain in writing how you feel than on the phone.“

(female, 28 years old, without migrant background)

### 3.2. Cultural Differences (Attitudes and Gender Differences)

There are culturally colored views on mental disorders that do not see any disease value in the symptoms. Above all, the picture changes depending on the gender of the person concerned:

“Again, about this thing in our home country, so I think in most countries, which is so Muslim, when a woman as an unmarried woman—if you are depressed or crying somehow—everyone says: ‘Yes, sure! She longs for a man.’ And then when she is married, everyone says, ‘Yes, she doesn’t like her husband. Apparently, she has a lover.’ In other words, it’s always viewed from a different perspective, a different position on the matter, and not what’s really there, what her problem is.”

(male, 23 years old, with migration background and refugee experience)

Concerning men, the picture is as follows:

“So they say, ‘Oh God, the poor man has worked so hard and suffered so much! He took care of the whole family and made sure they got something to eat. Of course you go crazy.’ Yes, with men it’s always a matter of course, ‘Oh, the poor guy!’, and with women, ‘Are you in love? Well, are you longing for a man? Would you like a man right away?’ Or, if you already have a man, ‘Well, are you in love with his brother?’ Right, it’s always like that. They don’t realize what their problems really are.”

(male, 23 years old, with migration background and refugee experience)

About the belief in “evil eyes” or Jinn as a cause of illness

“So there is a girl, she is about 25 years old. She has already studied the whole Koran. She was finished and took several courses, so… language courses. And has her diploma and everything.… At that age it’s marriageable age there.… She still hasn’t married. And she was acting a little bit strange. And all her parents and her family, her neighbors see her as mentally ill because she behaves a little bit different. And they say okay, I say ‘eye’ or it’s magic, witchcraft. Or because she didn’t marry… that she has a jinn in her body.”

(female, 20 years old, with migrant background)

### 3.3. Media Use in the Context of Mental Health Problems

The participants reported extensively on various media that are an integral part of their everyday lives. Reasons for media use were mentioned: Learning/researching; leisure time, including entertainment, relaxation and pursuing interests; communication/networking; being able to keep up or be up-to-date; practical help in everyday life. In the context of psyche, self-diagnosis or diagnosis for others was also mentioned.

#### 3.3.1. Search Strategies

First of all, it was emphasized that it is difficult to obtain reliable information and that it always involves an effort. This contributes to the fact that one does not search at all and puts back his needs and complaints:

“Sometimes there is the short thought, ‘I need help’, but then it’s gone again. The hurdle is too big, to get information would be too much effort.”

(female, 23 years old, without migration background)

Basically, searches in search engines are viewed critically and as something that is difficult:

“When searching on google, the problem is that google shows those things first an average user concerns most or what has been viewed most. This means, when starting something new you need the help of our main media providers for advertisement to be found.”

(male, 22 years old, without migration background)

In general, participants reported that they explicitly call up pages where it is evident that professionals, e.g., doctors, produce content or respond to questions, but do not feel addressed:

“Well, I first looked for the diagnosis and then for what was available on the Internet. And then there were sites like Netdoktor or Onmeda. There they focus on medication, on symptoms and then at the bottom they provide a reference: So that you can contact a specialist or something like that. There also exist self-help groups and stuff like that. But, yes it wasn’t really personal. So I did not feel addressed.”

(male, 20 years old, with migration background)

Various problems were raised in the search for help. For example, the fundamental doubts as to whether one’s own research and assessments are correct. These doubts then lead to further obstacles “Am I allowed to take advantage of this now at all? What if I misdiagnosed myself?” A further problem is that the technical language would be a deterrent. The participants reported that they did not know who was helping with which problem. At this point, participants with a migrant background mentioned that there can be elementary difficulties when filling out the questionnaires.

Some participants mentioned that things “you just need to know” are not communicated (such as the possibility of four “trial” sessions before starting therapy). Furthermore, there are no helpful hints, only many telephone numbers, which implies a lot of self-initiative is needed.

Many participants also mentioned that “Dr. Google” should be avoided. Looking for second opinions on the net would only confuse and instill fear

From the perspective of the helping person it is difficult to point the person concerned to the appropriate offer—”No one can be expected to be consolidated as a caregiver/reference person.”

#### 3.3.2. Needs

Various needs were expressed regarding media content in general and the website on the subject of psyche.

- First of all, that it has to be a serious offer with contents of concerned persons and professionals, which are personally presented. Because only personal stories from other affected people can help and inspire, but are not enough. A professional person has to provide content as well.
- The identification with the offer must be simple, the question, “Am I right here?” should be answered directly.
- The offering should motivate people to move out of the digital world and initiate personal meetings. There should be a very clear indication that this is no substitute for therapy. This should pave the way to therapy.
- A critical enlightenment is seen as advantageous, especially that a demarcation from and esotericism takes place, whereby mindfulness is especially emphasized as clearly helpful.
- The use of questionnaires or checklists was affirmed: Is help needed? If so, which form? Possible diagnosis?
- Videos are interesting for the target group, it was stressed that they should be short. Texts are preferred because they can be skimmed over independently and quickly classified.
- A quiz, on the other hand, is viewed rather critically (“What’s the point?”; the topic is too serious for a game)
- Statistics can show (if they are chosen well): I am not alone in this.
- Important facts should be pointed out: What can I do? How long does it take? Where can I find help? What are the consequences? What can positive prospects look like?
- When linking to regional offers sufficient information is very important!
- It is important to remember that telephoning can be difficult or that the surroundings of an institution or how to get there can be crucial for the decision for or against this institution.

A lack of knowledge about the content and process of a therapy was expressed:

“My sister already had therapeutic treatments.… And I ask myself, What is actually discussed in these hours? Is there a dialogue or a monologue taking place?”

(female, 28 years old, without migration background)

#### 3.3.3. Quality Factors of Websites

Seriousness is recognized very differently. There were participants who based the trustworthiness of a site on how many people use the site and how well known the offer is: “The more people use it, the greater my trust in it”. Additionally, decisive for some is the look of the page/app. As long as a professional design can be recognized, such as a reasonable structure and coherent menu, no advertising or annoying banners/popups and the information about the operator and promoter of the digital offer, trust is placed. The design for a serious-looking site is coherently described by the participants: clear, “good” design; no frills; after all, it is a serious offer; little distraction (“I have never watched a video on a website before”) and a trustworthy choice of color. Some participants demonstrated very good media skills, which ensured that they were able to assess the quality of the content in a much more differentiated way.

#### 3.3.4. Impact/Benefit of a Website

The idea was put forward that, taking into account the fact that some families have no understanding of mental health problems and that there are also care deficits in the health care system, online services could act as an interim solution.

“Okay I don’t have this backbone at home. For whatever reasons, you should at least have the possibility to get help elsewhere if you want to. If it is not given at home. Because I think you have to get help somewhere and I see it as a must. So as I said, this is not only a privilege, it should really be part of it like breathing, eating, sleeping. Just like first aid. If I feel bad, I call an ambulance. That is with us if I feel bad, I call the ambulance. And I think this help… should not only be physical help, but also mental help.”

(female, 28 years old, without migration background)

Barriers when talking to your parents/others about mental problems and anticipated benefits a website would hold:

“That you even open up a third section where the children really say “Mom, watch out. Ehm maybe you don’t quite understand it all. Here is this page. You can simply write it down, give it to them and the parents will be able to visit this page. The page alone will be very meaningful and afterwards, when the parents have read it, they might be like, ‘aha my daughter or my son is not the only one. And there seems to be something wrong.’ This way you have the possibility to communicate with your parents. It’s sad when you don’t have it face to face.… But maybe this is the first step.”

(female, 28 years old, without migration background)

#### 3.3.5. Advantages and Risks of a Diagnosis

The question of the advantages or disadvantages of remote diagnosis (self vs. others) via the Internet was extensively and controversially discussed.

As advantage was argued:

“In my opinion, diagnoses do have a value. They can indeed be helpful. At first you don’t know what your problem is, and therefore don’t know how to go against it. You simply don’t know where to start. The diagnosis gives you a name to work with. Now you can start to get rid of it. That’s why it is a good starting point. If you don’t understand what’s wrong, you first need to identify the basic problem, which is a problem in itself.”

(male, 25 years old, with migration background)

As disadvantage was argued:

“I also think so. In my opinion, it is very, very dangerous to only use the internet, especially when you immediately trust everything you read. When it comes to mental problems it can be very complex and experts are needed to watch over it. You can’t try to identify and understand every term by yourself, just to estimate your own position. I don’t like that. Most of all, if you do it this way, you automatically apply a negative identity to yourself and get into it way too much. You could develop even more symptoms, just because they fit the diagnosis you just found. Your behavior will change depending on the illness you think you have.”

(female, 24 years old, with migration background)

#### 3.3.6. Disturbing or Triggering Contents and Responses

Experiences were reported on media content having very disturbing effects. It was mentioned that these are dangers that can often be found on the Internet. Something like romanticizing suicide; or encouraging each other to unhealthy behavior (e.g., anorexia) or self-harming behavior.

“I think that really was the worst thing I could do at that time…. Well, my friends and others teased me in school always saying that I’m depressive. That was when I first entered the word depression into Tumblr. Immediately images of arms that where cut open appeared. Below it where words like, ‘It did so good. I feel better I’m not sad anymore, the pressure’s off.’ And as a 14-year-old, when you look at something like that, you think, ‘Ah okay, maybe it’ll help me. I’ve been struggling with this for so long. This is the way out.’ Now when you’re a little bit older of course you think differently, but when you’re that deep in it at that age, everything pulls you along. Today I would do it completely different… accordingly I would not even enter something like that. Especially not on pages like Instagram, Tumblr, Facebook, or such. Even on Google. If you type in depression and go to pictures, you will immediately get such pictures of self-harm. Immediately. You won’t get a table with things you can do to distract yourself or with skills that help you if there is the pressure to hurt yourself. There are only these pictures of self-harm. Pictures of people crying. Things like that.”

(female, 18 years old, with migration background)

## 4. Discussion

Mental disorders already manifest themselves in adolescence and young adulthood and persist into adulthood or represent preliminary stages of initial manifestation [52,53]. In accordance with WHO [15] findings, our research showed that adolescents and young adults, despite their young age, already have mental health experiences and problems. In particular, severe mental disorders such as bipolar disorders, depression, alcohol-related disorders, and schizophrenia have an early age of manifestation [53].

Some participants of the FGD expressed the need that the topic of mental health should be included in the life course, i.e., should be dealt with at school, if not in kindergarten. Stigmatizations related to mental illness are still present [10] and interventions to reduce it exist [54]. Empirical findings show that young people in particular experience stigmatization and shame, problems in recognizing symptoms in the sense of low MHL and a preference for autonomy in coping with mental health problems as the main obstacles to seeking help [55]. Studies show that mental health, help-seeking efficiency, and positive attitudes towards people with mental illness can be strengthened by promoting MHL [28,55,56,57,58]. For example, through the targeted promotion of mental health competence in the form of training, the quality of the intention to help depressed persons, and the self-confidence in providing help could be improved. Likewise the stigmatization and the desire for social distance towards depressive persons decreased [59]. Another study showed that increased knowledge about schizophrenia combined with increased personal social contact was associated with improved attitudes toward people with schizophrenia [60]. In summary, the findings indicate that MHL is related to health and social outcomes. In addition, MHL can be promoted in a targeted manner through interventions. A stronger focus on MHL among others in prevention can reduce health inequalities [61,62].

### 4.1. Mental Illness and Culture

There are multiple definitions of a migration background, either depending on nationality, migration, or both. Categorizations are generally problematic, as they can be stigmatizing and may lead to exclusions [63,64]. Evidence of experiences of discrimination in the education system, in vocational training, in the media, in the housing market in the context of migration background is available [64]. The refugees in particular are facing many challenges. Young people with a history of migration are a population that has to acquire skills for themselves and work their way through systems. They often act as translators for their parents, in everyday life and also during visits to the doctor [65], which is why promoting their health literacy is particularly crucial for the entire migrant population. However, in Germany in particular there is a lack of data on Health literacy among migrants [66].

Research on Turkish migrants in Germany has shown that the first generation of migrants of Turkish origin has a low sociodemographic and socioeconomic status and has more psychological problems than the native Germans. The first generation has higher rates of depression, panic attacks, and suicidal thoughts, while the second generation has better sociodemographic characteristics and comparable psychological problems to native Germans [67]. In particular, some migrant populations, such as those of Turkish origin, suffer more from mental disorders than the general population [68]. At the same time, there is evidence that the motivation for psychotherapy in the group of Turkish immigrants in Germany is low and that psychotherapeutic treatment in this subgroup is not as effective as in the general German population [68].

In keeping with the experiences and observations of young people with a migrant background reported in this study—especially in the FGD, which took place outside the school context—the Muslim faith in this case has an influence on the view of illness. It became apparent that, in connection with religion, the notions of illness with regard to mental health problems varied from those of the non-Muslim participants in the other focus groups in the schools.

The authors Laabdallaoui and Rüschoff [69], both working in psychiatric–psychotherapeutic fields, summarized on the basis of religious sources what is considered to cause illness according to Islam. In addition to the medical causes of illness, there are other triggers such as the “evil eye”, magic and other spirit beings, the so-called “jinn”, which are to be understood as follows:

- Faith in the effect of the evil eye

Accordingly, Muslims believe that the gaze of a person can have a negative effect and can harm the person opposite. The evil eye is said to cause any kind of illness, but depressive symptoms, tension, constant yawning and general malaise are attributed to the effect of the evil eye, to which one is at the mercy of.

- Belief in the effect of magic or sorcery

Abusive practice, which is strictly forbidden in religion, but is nevertheless quite present. Particularly in the case of mental and psychiatric illnesses, the possibility of a cause by magic is not excluded

- Faith in the effect of other beings, the jinn

There is a belief that when jinn are disturbed in their tranquility, they can cause a disturbance to humans [61,70]. In particular with mental illnesses, psychosis fainting fits, and paralysis phenomena an obsession by a jinn is assumed [71].

Against the background of these ideas of illness, in the case of mental illness the affected persons or their relatives additionally consult a religious scholar, imam, or hodja, who is supposed to support the healing process through Koran recitations or special invocations [69,72]. According to Laabdallaoui and Rüschoff (2010), knowledge of the presumed subjective causes of disease is of great importance for the success of treatment [69].

### 4.2. Mental Illness and Gender

In the study conducted, it was noticed that female participants are more involved in the mental health issues of their relatives and thus have more experiences with mental illnesses. Furthermore, perceptions, attitudes, prevalence, and management of mental illness differ between men and women. Young women have a higher prevalence and a higher severity of depressive symptoms. Brettschneider et al. (2018) investigated the prevalence of depression in the German population and underlined that the 12-month prevalence in women aged 18–34 years rose by about twice as much in the years 1997–1999 and 2009–2012 from 8.8% to 15.6%, although the prevalence of depression in the general population did not increase. The conclusion is that an increased distribution of prevalence among young women is a risk in this specific subgroup [23].

Other research results show that depression in adolescence entails a greater risk of suicidal tendencies among young women; those affected conduct more suicide attempts than men [73]. However, suicide attempts are more often fatal for men [74]. Another gender difference relates to the symptoms of depression: women show typical symptoms such as sadness, withdrawal, increased appetite, weight gain, sleep disorders, somatoform disorders, increased crying, and feelings of guilt. They report more frequent and more pronounced symptoms than men, while men more often suffer from health problems, insomnia, overall listlessness and agitation [75].

Migration, acculturation and mental disorders cause different stress and adaptation processes in men and women. It was found that female migrants with depression or anxiety disorders have higher levels of the migration-related stressor than male migrants, i.e., stress associated with migration and acculturation. In addition, feelings of guilt and self-condemnation are stronger and more often pronounced among female migrants than among male migrants and native Germans. These two manifestations could be related to mental disorders [76]. In this context the term acculturation is understood as a process of cultural identity adaptation when there is long-term contact between members of two different cultures. Morawa and Erim (2014) divide acculturation into four components: integration, assimilation, separation, and marginalization [77]. They tested correlations to depression symptoms with the result of integration being the lowest and marginalization the highest correlated. In contrast to previously stated studies, gender had no influence on acculturation, but on depressive symptoms. Women have a higher level of depressive symptoms than men. Moreover, the first generation of migrants shows more depressive symptoms than the second [77].

Among migrants of Turkish origin, stigma is associated with depression and psychological stress. Patients who are more depressed and show a higher level of psychological stress experience their condition even more stigmatized. Depression and symptoms of other mental disorders influence the concerns and complaints of stigma, which results in even more or stronger concerns. The fear of stigma can lead to misunderstandings, wrong diagnoses and renewed stigmatization [78].

Depending on whether woman or a men is affected in mental illness, there may be general culture-dependent differences in the way people deal with it, what shapes understanding and acceptance (see results).

### 4.3. New Website to be Developed

As became clear from the focus group discussions, the media play an important role in the lives of young people. They use the internet for a variety of purposes and see many advantages in it, for example in the context of school for learning purposes, but also for researching things that concern them personally. The following study also showed the role of the media: An online survey on “COVID-19 Health Literacy” (COVID-HL), in which 14,895 students from 130 universities in Germany participated, focused on the evaluation of online health information, digital health literacy, and mental health [79]. Results show that the Internet has a special significance as a source of information and that every fifth student has already looked for information on dealing with mental stress. Students found it difficult to judge how reliable the health information on the Web is [79].

The observation that media are very important in the lives of young people has led to the proposal to include the aspect of media literacy in the model of health literacy especially among young people [25]. From the statements of the participants, it became clear that existing offers/possibilities were not confidence-inspiring enough. According to this, a special offer should be created for this purpose, as the existing offers are not sufficiently target-oriented or are not adapted to the target group and its wishes.

In order to overcome language barriers, graphic and audio-visual content accompanying the text should be integrated in addition to a simple yet comprehensive provision of information as text [80,81]. Graphs can be used to illustrate both complex issues and the fundamental importance of mental health in society—an under-represented topic compared to physical health in traditional media, leading to a distorted picture of the relevance, distribution and impact of mental illness [82]. Audiovisual content also has an increased effect on the recipients’ motivation to deal with the topic, to question their own behavior and to integrate mental health promotion measures into their everyday lives [83]. The gap between the intended and actual future behavior of the recipients can be closed by using understandable, positive language in the videos [84], so that in addition to the increase in knowledge, the goal of removing taboos and stigmatization is achieved.

Interactive design allows to guide the recipients through the website and to provide information at the right time and in the right amount. This principle is considered a factor for a successful improvement of health literacy [85,86]. Insights into production and reception as well as long-term changes in attitudes and actions are considered to be insufficiently researched [87], and can also be gained through the planned evaluation.

The destigmatization of mental illness also plays a central role in the preparation of all information and topic-specific content and is pursued through the use of target group-specific language and education. Prejudices and stigma are lowered by the fact that a broad and well-founded education takes place and the focus is not on warning/fear appeals, but on serious and realistic advice. The innovation and model character of the project lies in the fact that a broad group (age, gender, migration background, and educational level) of teenagers and young people can be reached with the digital offer. Due to the multilingualism, particularly vulnerable groups are taken into account.

Through cooperation with the associated partners and references to the project on their websites as well as social media channels, the website is to be made widely known on a long-term basis. A long-term use and perpetuation of the project is guaranteed by the cooperation with the associated partners in the schools, therapy institutes, and migrant organizations, thus covering the target groups and the typical contact points and ensuring that the existing offer continuously reaches the target group.

#### 4.3.1. Properties of the website to be developed/Elements of the website

Based on the results from the FGD, the following elements will be implemented on the website:

1. To improve the mediation of psychological content, which can be quite complex, multimodal processing is necessary. This made it clear that texts and videos are in demand within the target group. Explicitly redundant content, which is, however, presented multimodally—i.e., via different media formats—is desired. For this reason, video clips are to be created with experts who convey complicated content in a comprehensible manner, thus supporting the texts on the website. For each presented disorder, especially depression, anxiety disorders, personality disorders, psychoses and addictions and possibly other disorders (e.g., eating disorders and obsessive-compulsive disorders), several short videos will be produced on the aspects of symptoms, causes, handling, and therapy.
2. Another result of the FGD is the demand for graphics. These are to be produced to present complex contents in a simplified way. Just like the videos, they serve to support the text-based content and are created and integrated according to the layout of the site.
3. Presentation of all contact points for mentally ill persons and their relatives: The goal is to present the offers on the website in a kind of “profile”, if necessary with photos of the contact points and/or the respective contact persons. In the FGD it became clear that existing information on the Internet was not manageable and could not be checked for seriousness; besides, the needs or hardships of people with acute psychological problems were not addressed.
4. During the FGD, the target group expressed the wish for an English version of the website. Here, participants with refugee experience identified English as a common and largely understandable language among young people. Therefore, the content should be provided in English. The information on the website will be kept identical for all. In line with the long-term project goals, it is planned to translate the content into Arabic and, if necessary, Turkish. The aim is to make the necessary information regarding mental health and illness equally accessible to people with and without language barriers.

In detail, the website aims to enable young people to obtain information quickly and easily about:

- What is the problem/burden/illness? (Description of the symptoms)
- How do you recognize this? (Diagnosis)
- How do you treat this? (Showing the treatment options/ways and contact persons)
- What does “mental health” mean and how can I strengthen my resilience? (Integration into the living reality of the target group, education, and practical tips).

#### 4.3.2. Aspects of the evaluation of the new website

To measure MHL, Jorm et al. (1997) used short vignettes describing a person suffering from a mental disorder (depression and schizophrenia). Subsequently, subjects were asked questions about their knowledge and attitudes to aspects of MHL, including helpful treatment, likely prognosis, risk factors and stigma [36]. The method of recording MHL via short vignettes described above is the most popular recording method, but it is criticized because the vignettes can only cover a small number of disorders and not all areas of MHL [28]. It is also argued that the use of vignettes neglects the ability to distinguish mental disorders from a mental health problem in the sense of everyday stress (Kutcher et al., 2016). In addition, reference is made to the limited generalizability and replicability of the findings due to the use of different and mostly culture-bound vignettes [88]. As part of the evaluation of the new website, the acceptance of the website in terms of design, layout, structure, and comprehensibility is to be recorded with young people with and without a migration background. This will be done by a combination of qualitative interviews and quantitative questionnaires. Overall, a recursive approach is needed, intermediate products are evaluated and adjusted, which requires further evaluation to check whether the changes were in the interest of the target group. In addition, the website’s contribution to increasing the level of information is to be recorded in a pre-post comparison. For this purpose, a small sample of young people should be asked to write down their knowledge about disorders and their most relevant parts (pre-test), then the website should be presented. After reception a new request for relevant information should be made (post-test), so that a possible increase in knowledge can be quantified. Finally, these results will be incorporated into the improved version of the website.

### 4.4. Limitations

One limitation of this study is that the nine focus groups were implemented in Cologne and the surrounding area and that a local dependency may exist with regard to the experiences in everyday life or in society as well as the view of the care situation. However, since it was not the aim of the study to achieve representative results or even generalizations to the target group of young people with and without a migration background, the local restriction has no significance. After all, the goal of qualitative research is to capture the participants’ individual perspectives on a particular topic and therefore serves as a means to better understand phenomena in a holistic manner [89]. Overall, each of the focus groups was uniquely constructed in its composition. The focus groups varied in terms of age, migration background, gender and religion of the participants.

There could be a selection bias here, as participants were recruited via announcements posted at schools and clubs, whereupon interested individuals could sign up.

It is possible that those who had a particular concern or interest in this topic and were willing to participate in the discussion within the group setting were more likely to do so. In contrast, there may be individuals who were affected by mental health issues and may have felt more deterred to participate in a group discussion on this sensitive topic. It could therefore be that possibly relevant aspects of affected persons, which would have been substantial to explore the topic, are not reflected in the material.

Another limitation may be the low variation in school types. Both schools that participated in our project—a total of five FGDs—were educational institutions that offer adults the opportunity to obtain a technical college entrance qualification and a general higher education entrance qualification via the second educational pathway. Originally, secondary schools from the ninth grade (15 and 16-year-old students) were also to be involved. However, during the course of the project, it became apparent that the requested schools were unable to participate in our study due to their own capacity limitations. Moreover, due to the current pandemic (COVID-19), no additional schools could be recruited. Consequently, for the purpose of feasibility in terms of schools, we limited ourselves to those schools with willingness to participate with adult students. However, this implies that the perspective of adolescents may have been somewhat neglected, despite the fact that there were also adolescent participants from clubs in our study.

## 5. Conclusions

Our research results show how widespread experiences with mental illness are in the target group of adolescents and young adults with and without migration background, who formed the study sample. It also became clear that media are indispensable in the everyday life of the participating young people and that health information on the Internet is an important resource for understanding the problem and finding solutions. With regard to the difficulties in handling and treatment, it became clear that there are very basic barriers to addressing psychological problems openly, since mental illness is still met with incomprehension and prejudice. In addition to these problems on the individual level, there are also deficits in health care, such as long waiting times for a place on a treatment program, which affect the society as a whole and make it difficult to receive timely and appropriate help. Young refugees in this sample are exposed to further barriers and care deficits in addition to the stresses and strains of fleeing, as they do not receive adequate help due to language-barriers and treatment options are hardly available. In the long term, these care deficits lead to chronification and irreversible consequences such as suicide. Adolescents and young adults with a migrant background (participants of the FGD 1, 7 and 8), who are now living in Germany in the third generation, also reported experiences of discrimination and further burdens. Muslim participants of this study reported a culturally colored view of mental illness.

It also became clear that young people want more openness and reliable information. The need to implement the topic of mental health into the education system on an institutional level was expressed. The need for reliable information on the Internet to help in an acute situation—at least as an interim solution—was also expressed. At the same time, negative aspects and dangers of media content were noted and clear indicators/markers of quality, reliability and professionalism, i.e., trustworthiness, were called for.

In summary, there is potential for the media to communicate this important topic of mental health in a sensitive, target group-oriented and professional manner. This could give rise to approaches in the health care system to focus even more strongly on this topic and to address the deficits in mental health care. At the societal level, raising awareness for mental illness could be made an integral part of the curriculum in educational institutions and barriers could be broken down. Further research is needed on young people’s perspective—especially the perspective of those with a migration background—on mental illness and the evaluation of existing offerings on the Internet.

## 6. Patents

This section is not mandatory, but may be added if there are patents resulting from the work reported in this manuscript.

## Figures and Tables

**Table 1:** Main characteristics of the participants in the Focus Group Discussions (FGD) (*N* = 68).

Gender		Male (*n*)	Female (*n*)
Age (years)	≤14	/	1
	15–19	2	8
	20–24	17	19
	25–29	5	15
	≥30	1	/
Migrant background	migrant background	13	27
	no migrant background	12	16
Total		25	43

**Table 2:** The first two levels of the category system.

Main Categories	Subcategories
(Mental health) challenges in everyday life	Experience and dealing with psychological problems
	Level of knowledge and attitudes towards mental health/illness
	Health care situation
	Reasons/Triggers for psychological problems
	Psychological symptoms/disorders
	Gender aspects
Health Literacy Components	Access Health Informations
	Understand Health Informations
	Appraise Health Informations
	Apply Health Informations
Digital media in everyday life	User interface/application purpose of end devices
	Types of media
	Duration of media use
	Reasons for media use
	Quality features
	Positive aspects of media
	Negative aspects of media
Transmission of health information (formats)	Communication types
	(Online-) Formats
	Used (foreign) language
	Requirements for the planned website (of the project)

## Data Availability

Data sharing not applicable.
